# Supplementary material for: Risk Factors for Emergency Department Unscheduled Return Visits
Source: Medicina (Kaunas). 2019 Aug 9;55(8):457. doi: 10.3390/medicina55080457 (PMC6723936; doi:10.3390/medicina55080457)
Supplement: Supplementary file 1 [file medicina-55-00457-s001.pdf]

Table S1. Groupings of presenting complaints.

Grouping of complaints	Presenting complaints (non-exhaustive)	Examples of eventual diagnoses
Abdominal pain	Localized abdominal pain<br>Generalized abdominal pain<br>Abdominal colic<br>Dyspepsia	Intestinal obstruction<br>Peptic ulcer disease<br>Acute appendicitis<br>Biliary colic
Fever	Patient-documented temperature or temperature ≥ 38.0°C at the ED with no localizing source	Viral fever<br>Neutropenic fever
Gastrointestinal	Nausea<br>Vomiting<br>Diarrhea<br>Constipation	Gastroenteritis<br>Inflammatory bowel disease<br>Irritable bowel syndrome
Upper respiratory tract infection	Cough<br>Rhinorrhea<br>Sore throat	Pharyngitis<br>Tonsillitis<br>Sinusitis
Musculoskeletal pain	Body aches<br>Joint pains	Osteoarthritis<br>Rheumatoid arthritis<br>Gout flare<br>Tendinitis
Trauma	Fall<br>Burns<br>Cut wound<br>Motor vehicle accident<br>Minor head injury	Abrasion<br>Contusion<br>Fracture<br>Dislocation<br>Concussion
Headache	Localized headache (e.g. bitemporal, occipital)<br>Generalized headache	Primary headache (e.g. migraine)<br>Intracranial bleed<br>Brain tumor
Lower back pain and sciatica	Backache<br>Radiation of pain to lower limb	Prolapsed intervertebral disc<br>Lumbar spondylosis

		Degenerative disc disease
Chest pain	Angina<br>Pleuritic chest pain	Acute myocardial infarction<br>Spontaneous pneumothorax<br>Atypical chest pain
Giddiness and vertigo	Dizziness (non-vertiginous)<br>Vertigo	Vestibular neuritis<br>Benign paroxysmal positional vertigo
Ophthalmological and otolaryngological	Eye redness, itch, floaters<br>Ear blockage, pain, discharge<br>Foreign body in eye or ear	Conjunctivitis<br>Subconjunctival hemorrhage<br>Otitis media, otitis externa<br>Cornea abrasion
Shortness of breath (excluding asthma)	Dyspnea	Acute bronchitis<br>Hyperventilation syndrome<br>Chronic obstructive lung disease
Urological	Dysuria<br>Hematuria<br>Urinary frequency, urgency<br>Urinary retention	Urinary tract infection<br>Benign prostatic hyperplasia<br>Blocked indwelling catheter
Dermatological	Generalized exanthem<br>Localized rash<br>Pruritus	Atopic dermatitis<br>Varicella<br>Urticaria
Renal colic	Flank pain<br>Pain radiating to groin	Ureteric stones
Obstetric and gynecological	Dysmenorrhea<br>Menorrhagia<br>Abnormal vaginal discharge<br>Vulvar mass<br>Per-vaginal bleeding in pregnancy	Endometriosis<br>Uterine fibroid<br>Pelvic inflammatory disease<br>Bartholin's cyst infection<br>Threatened miscarriage
Cellulitis and abscess	Limb redness and swelling<br>Painful skin lump	Cellulitis<br>Abscess

	Insect or animal bites	Bite wounds
Neurological deficit	Loss of consciousness	Vasovagal syncope
	Weakness	Transient ischemic attack
	Numbness, paresthesia	Carpal tunnel syndrome
Psychiatric	Depressed mood	Acute stress reaction
	Anxiety	Depressive disorder
	Insomnia	Anxiety disorder
	Hallucination	Schizophrenia
Asthma	Wheezing	Asthma exacerbation
	Shortness of breath, cough (on background of asthma)	
Others	Palpitations	Thyrotoxicosis
	Testicular pain	Epididymo-orchitis
	Post-operative complications	Dislodged nephrostomy tube
